# Publisher Correction: Transformers significantly improve splice site prediction

**DOI:** 10.1038/s42003-024-07379-9

**Published:** 2024-12-18

**Authors:** Benedikt A. Jónsson, Gísli H. Halldórsson, Steinþór Árdal, Sölvi Rögnvaldsson, Eyþór Einarsson, Patrick Sulem, Daníel F. Guðbjartsson, Páll Melsted, Kári Stefánsson, Magnús Ö. Úlfarsson

**Affiliations:** 1 deCODE Genetics/Amgen Inc., Reykjavik, Iceland; 2 University of Iceland, Reykjavik, Iceland

**Keywords:** RNA splicing, Machine learning

Correction to: *Communications Biology* 10.1038/s42003-024-07298-9, published online 4 December 2024

In Table 2 footnote, the sentence beginning with “The performance metrics are…” in this article, “cross-entropy,” was incorrectly captured, it should have read “The performance metrics are PR-AUC and top-k accuracy.

