# Supplementary material for: Transformers significantly improve splice site prediction
Source: Commun Biol. 2024 Dec 4;7:1616. doi: 10.1038/s42003-024-07298-9 (PMC11618611; doi:10.1038/s42003-024-07298-9)
Supplement: Supplementary file 2 — Supplementary information [file 42003_2024_7298_MOESM2_ESM.pdf]

Supplementary Information

Model Architecture Diagrams

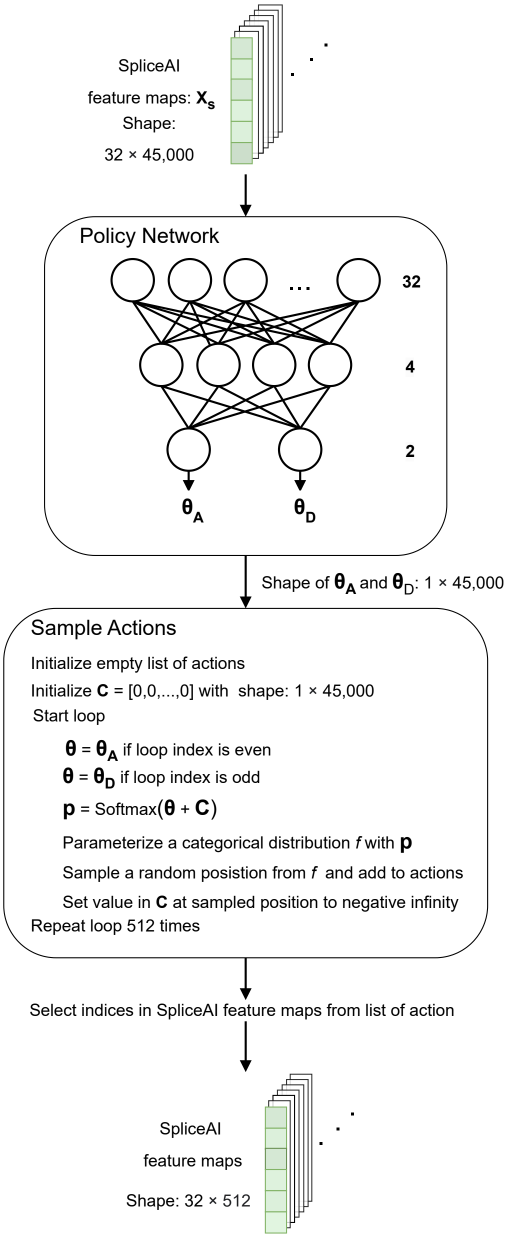

Figure S1: Components of the splice site selection module.

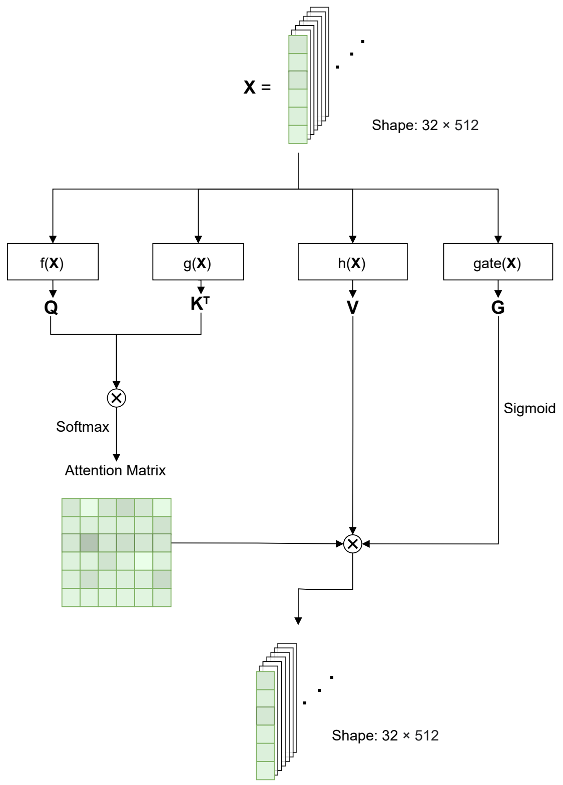

Figure S2: Components of the gated multi-headed attention layer.

PR-AUC by splice site type

Table S1 shows the PR-AUC results for ENSEMBL and GENCODE annotations, previously seen in Figure 1, split by splice site type (acceptor and donor). These models are all slightly more accurate at predicting donors than acceptors. The table also shows that using the pre-trained weights for SpliceAI-10k, made available by the authors, has the poorest performance. This drop in PR-AUC might be due to the weights being trained on a combined set of splice sites from GENCODE and GTEx.

GTEx classification results

The precision and recall of Transformer-45k for a few threshold values are shown in Table S2. The ROC and precision recall curves for the GTEx classification results (from Table 1) and the total number true positive and false positive splice sites as a function of the decision threshold are shown in Figure S3.

Validation on Spliceator data

Using the splice site annotations from the Spliceator method [1], we tested both Transformer-45k, with high recall thresholds 0.01 and 0.1, and the standard threshold 0.5. Our results show that using 0.01 as the threshold outperforms Spliceator with regard to every metric (Table S3).

Table S1: PR-AUC score for splice site, acceptors, and donors in GENCODE and ENSEMBL. Best score displayed in bold.

	ENSEMBL (N = 2×89,712)			GENCODE (N = 2×14,289)		
	Splice site PR-AUC	Acceptor PR-AUC	Donor PR-AUC	Splice site PR-AUC	Acceptor PR-AUC	Donor PR-AUC
Transformer-45k trained on ENSEMBL	**0.969**	**0.967**	**0.971**	**0.979**	**0.978**	**0.981**
Transformer-10k trained on ENSEMBL	0.966	0.964	0.968	0.978	0.977	0.979
SpliceAI-10k trained on ENSEMBL	0.965	0.964	0.967	0.977	0.976	0.978
SpliceAI-10k trained on GENCODE	0.963	0.960	0.966	0.975	0.974	0.977
SpliceAI-10k Pre-trained weights	0.955	0.951	0.958	0.962	0.959	0.965

Table S2: Precision and recall for fine-tuned Transformer-45k on GTEx V8 splice junctions, for five different decision thresholds.

Threshold	Precision	Recall
0.01	0.188	0.972
0.1	0.532	0.858
0.25	0.786	0.744
0.5	0.943	0.642
0.75	0.985	0.585

This dataset only includes a 600 nt context around each site, we expect that the performance of our model would be further improved if it had access to a large context.

Table S3: Performance metrics for Transformer-45k and Spliceator on the GS_1 dataset.

	Accuracy		Precision		Sensitivity		Specificity		F1 Score	
	Donor	Acceptor	Donor	Acceptor	Donor	Acceptor	Donor	Acceptor	Donor	Acceptor
Transformer-45k (0.01)	**96.99**	96.07	96.27	95.02	**97.76**	**97.31**	96.22	94.82	**97.01**	96.15
Transformer-45k (0.1)	96.85	**96.94**	98.75	98.37	94.88	95.50	98.80	98.40	96.78	**96.92**
Transformer-45k (0.5)	87.90	95.07	**99.67**	**99.61**	76.02	94.89	**99.75**	**98.80**	86.25	94.87
Spliceator	92.82	89.02	89.88	86.23	96.53	92.93	89.12	85.15	93.08	89.40

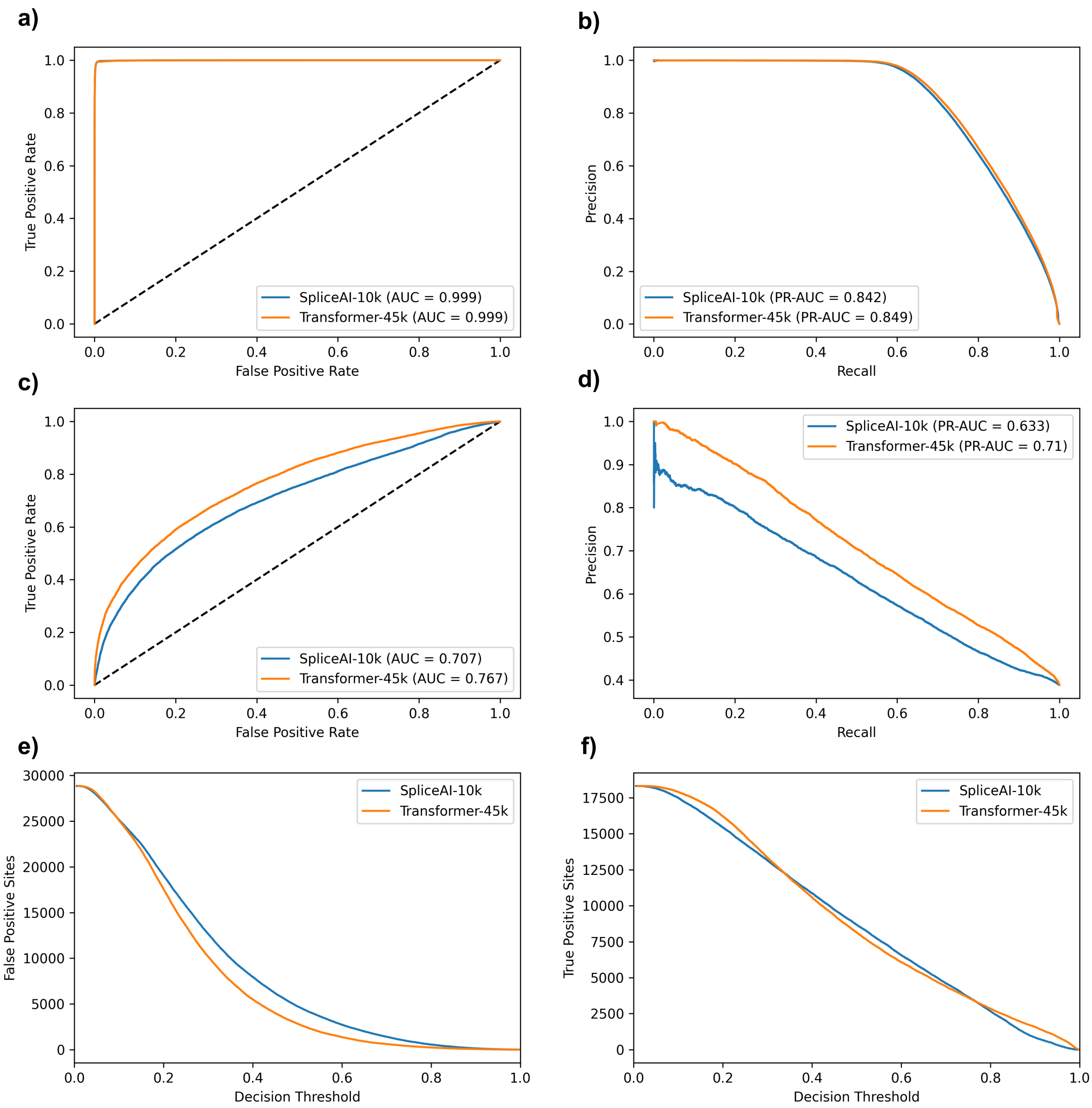

Figure S3: GTEx splice site classification results for Splice-10k and Transformer-45k.
a) ROC curve for SpliceAI-10k and Transformer-45k.
b) Precision recall curve curve for SpliceAI-10k and Transformer-45k.
c) ROC curve for cases where SpliceAI and Transformer-45k disagree (TVD ≥ 0.1).
d) Precision recall curve for cases where SpliceAI and Transformer-45k disagree (TVD ≥ 0.1).
e) The total number false positive splice sites as a function of the decision threshold for cases where SpliceAI and Transformer-45k disagree (TVD ≥ 0.1).
f) The total number true positive splice sites as a function of the decision threshold for cases where SpliceAI and Transformer-45k disagree (TVD ≥ 0.1).

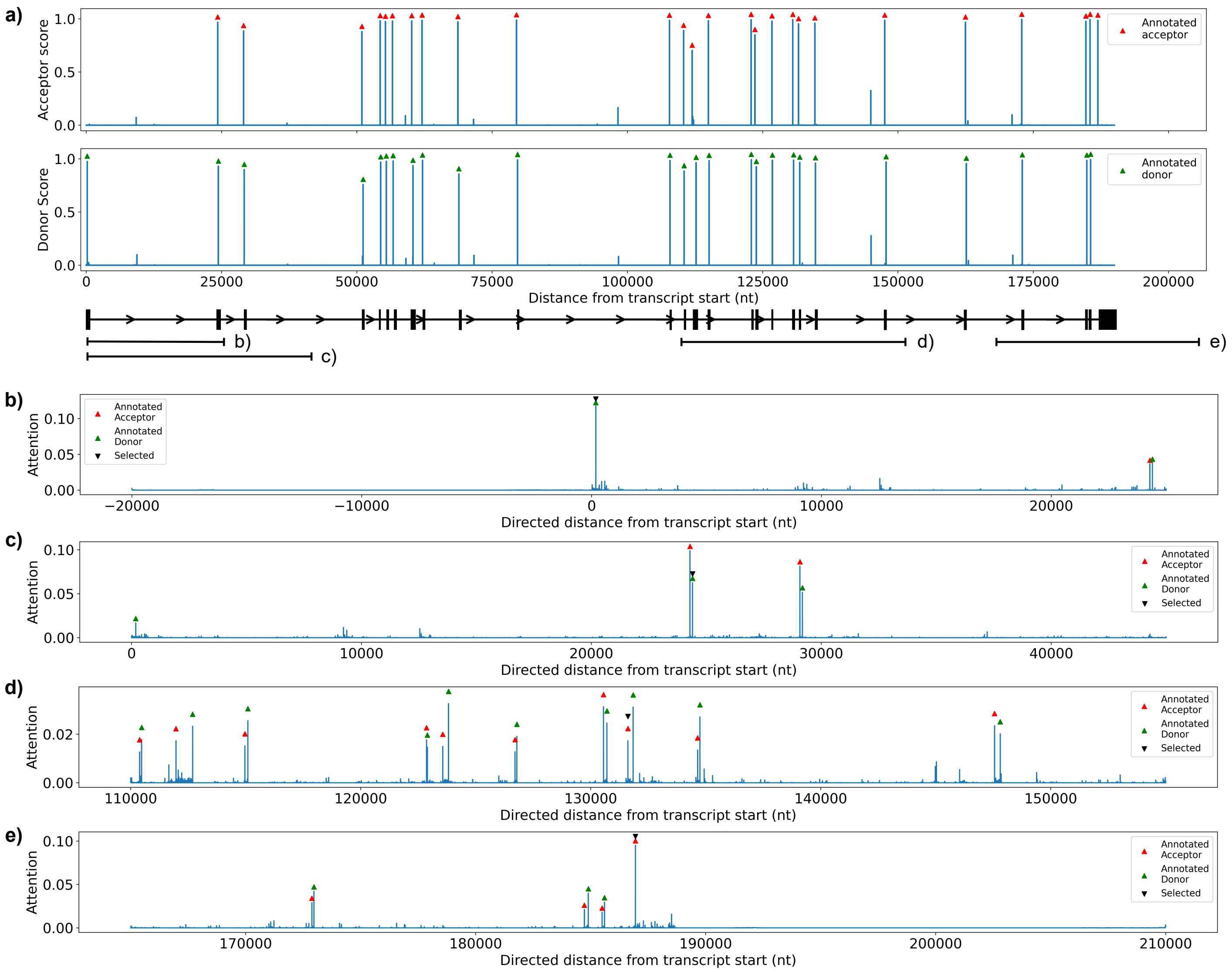

Figure S4: A closer look at splice site and attention scores in *CFTR*. Here we used the canonical transcript for *CFTR* (ENST00000003084), it has 26 splice-junctions (all detected by the model), length 188,702 nt, and it was not included in the training set.
a) Transformer-45k splice site scores for *CFTR*.
b) Transformer-45k attention scores for the donor at the start of *CFTR*.
c) Transformer-45k attention scores for the second donor in *CFTR*.
d) Transformer-45k attention scores for acceptor located ~135,000 nt from the start of *CFTR*.
e) Attention scores for the final acceptor in *CFTR*.

PCA of Penultimate Layer Embeddings

We looked at the nucleotide sequence for *CFTR* and extracted embeddings from the penultimate layer of Transformer-45k. We applied principal component analysis to the embeddings from the ten models and observed that the first two principal components (PCs) are correlated with the distance to the nearest splice site (PC_1: [$r_s = -0.573$, $p = 0$], and PC_2: [$r_s = -0.377$, $p = 0$]) (Figure S5**a**). The first two PCs explain 50.0% and 5.3% of the variance in the embeddings.

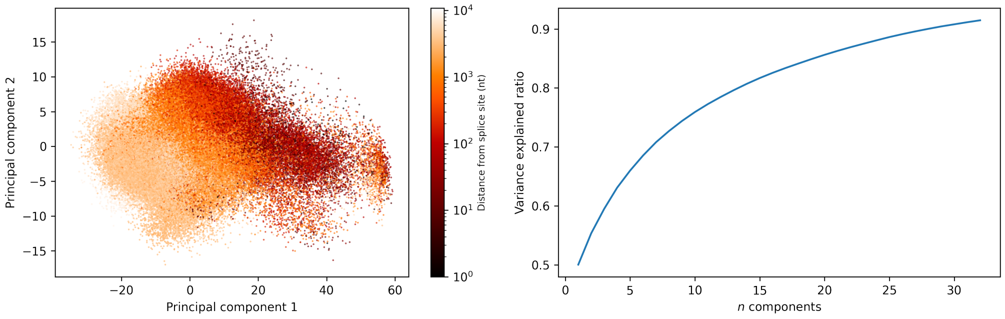

Figure S5: PCA of the *CFTR* gene sequence as encoded in the penultimate layer of Transformer-45k model. Plot on the left shows the first two principal components of the *CFTR* sequence and plot on the right show the cumulative variance explained ratio of the first 32 PCs.

References

[1] Nicolas Scalzitti, Arnaud Kress, Romain Orhand, Thomas Weber, Luc Moulinier, Anne Jeannin-Girardon, Pierre Collet, Olivier Poch, and Julie D Thompson. Spliceator: Multi-species splice site prediction using convolutional neural networks. *BMC bioinformatics*, 22(1):1–26, 2021.
