# Supplementary material for: Transformers significantly improve splice site prediction
Source: Commun Biol. 2024 Dec 4;7:1616. doi: 10.1038/s42003-024-07298-9 (PMC11618611; doi:10.1038/s42003-024-07298-9)
Supplement: Supplementary file 3 — reporting summary [file 42003_2024_7298_MOESM3_ESM.pdf]

nature portfolio

Corresponding author(s): M.Ö. Úlfarsson, K. Stefánsson

Last updated by author(s): 13/11/24

Reporting Summary

Nature Portfolio wishes to improve the reproducibility of the work that we publish. This form provides structure for consistency and transparency in reporting. For further information on Nature Portfolio policies, see our Editorial Policies and the Editorial Policy Checklist.

Statistics

For all statistical analyses, confirm that the following items are present in the figure legend, table legend, main text, or Methods section.

n/a	Confirmed	
☐	☒	The exact sample size (*n*) for each experimental group/condition, given as a discrete number and unit of measurement
☐	☒	A statement on whether measurements were taken from distinct samples or whether the same sample was measured repeatedly
☐	☒	The statistical test(s) used AND whether they are one- or two-sided<br>*Only common tests should be described solely by name; describe more complex techniques in the Methods section.*
☐	☒	A description of all covariates tested
☐	☒	A description of any assumptions or corrections, such as tests of normality and adjustment for multiple comparisons
☐	☒	A full description of the statistical parameters including central tendency (e.g. means) or other basic estimates (e.g. regression coefficient) AND variation (e.g. standard deviation) or associated estimates of uncertainty (e.g. confidence intervals)
☒	☐	For null hypothesis testing, the test statistic (e.g. *F*, *t*, *r*) with confidence intervals, effect sizes, degrees of freedom and *P* value noted<br>*Give P values as exact values whenever suitable.*
☒	☐	For Bayesian analysis, information on the choice of priors and Markov chain Monte Carlo settings
☒	☐	For hierarchical and complex designs, identification of the appropriate level for tests and full reporting of outcomes
☒	☐	Estimates of effect sizes (e.g. Cohen's *d*, Pearson's *r*), indicating how they were calculated

Our web collection on statistics for biologists contains articles on many of the points above.

Software and code

Policy information about availability of computer code

Data collection	Paired-end Poly-A mRNA RNA-Seq samples with read length 2x125 were collected in Iceland using Illumina NovaSeq and HiSeq machines.
Data analysis	RNA-Seq samples were aligned to the reference genome using STAR v2.5.3a. The code used to generate the results can be found here: https://github.com/benniatli/Spliceformer

For manuscripts utilizing custom algorithms or software that are central to the research but not yet described in published literature, software must be made available to editors and reviewers. We strongly encourage code deposition in a community repository (e.g. GitHub). See the Nature Portfolio guidelines for submitting code & software for further information.

Data

Policy information about availability of data

All manuscripts must include a data availability statement. This statement should provide the following information, where applicable:

- Accession codes, unique identifiers, or web links for publicly available datasets
- A description of any restrictions on data availability
- For clinical datasets or third party data, please ensure that the statement adheres to our policy

The data used in this study was generated from gene annotations obtained from ENSEMBL (http://www.ensembl.org/Homo_sapiens/Info/Index), GENCODE (https://www.gencodegenes.org/human/) and RNA-Seq data obtained through the GTEx Portal (https://gtexportal.org/home/datasets) and from an Icelandic cohort sequenced by deCODE genetics. ClinVar variants are available through https://ftp.ncbi.nlm.nih.gov/pub/clinvar/. The splice site annotations used for fine-tuning and

evaluating the models are included in Supplementary Table 1 (Icelandic whole blood combined with GTEx V8) and Supplementary Table 2 (GTEx V8 only). The Icelandic RNA-Seq data used in this study are not publicly available due to information, contained within them, that could compromise research participant privacy and releasing this information publicly would be against Icelandic state law. Other data supporting the findings of this study are available from the corresponding authors upon reasonable request.

Research involving human participants, their data, or biological material

Policy information about studies with human participants or human data. See also policy information about sex, gender (identity/presentation), and sexual orientation and race, ethnicity and racism.

Reporting on sex and gender	Sex determined from genotype data was considered as a covariate while adjusting percent spliced in (PSI) before the sQTL association scan. No other sex-based analysis was considered.
Reporting on race, ethnicity, or other socially relevant groupings	The cohort was a homogeneous population of Icelanders. In the sQTL association scan we adjusted for kinship since the pedigree of Icelanders was available.
Population characteristics	A cohort of 17,848 individuals from Iceland (9,784 females, 8,064 males). The year of birth (YOB) data available to us was binned into 5 year bins. The oldest male and female were born closest to 1920 and youngest male and female were born closest to 2005. The median YOB for both sexes was 1960.
Recruitment	*DNA and RNA isolated from whole blood was sequenced from 17,848 individuals participating in various studies at deCODE genetics.*
Ethics oversight	*This research received approval from the National Bioethics Committee of Iceland (approval number VSN 14-015) and was conducted in accordance with guidelines from the Icelandic Data Protection Authority (PV_2017060950PS/--)*

Note that full information on the approval of the study protocol must also be provided in the manuscript.

Field-specific reporting

Please select the one below that is the best fit for your research. If you are not sure, read the appropriate sections before making your selection.

☒ Life sciences ☐ Behavioural & social sciences ☐ Ecological, evolutionary & environmental sciences

For a reference copy of the document with all sections, see nature.com/documents/nr-reporting-summary-flat.pdf

Life sciences study design

All studies must disclose on these points even when the disclosure is negative.

Sample size	No sample size calculation was performed. The sample size used in this study was determined by the number of RNA sequenced samples when the study was initiated. Since we detected a large number of statistically significant SQTLs we can conclude that the sample size is sufficent.
Data exclusions	Splice junctions detected in the RNA-Seq samples were filtered out if the junction did not have reads in four or more subjects, either end of the junction is in a ENCODE blacklist region or a simple repeat region, and if the junctions were not apart of a LeafCutter clusters that includes canonical splice sites.
Replication	*The majority (94.2%) of lead sQTLs identified from whole blood in GTEx V8 were replicated in our cohort. All attempts at replication were successful.*
Randomization	*Not applicable. This was a cis-association study, not a randomized trial.*
Blinding	Not applicable. This was a cis-association study, not a randomized trial. No blinding was therefore required.

Reporting for specific materials, systems and methods

We require information from authors about some types of materials, experimental systems and methods used in many studies. Here, indicate whether each material, system or method listed is relevant to your study. If you are not sure if a list item applies to your research, read the appropriate section before selecting a response.

Materials & experimental systems

n/a	Involved in the study
☒	☐ Antibodies
☒	☐ Eukaryotic cell lines
☒	☐ Palaeontology and archaeology
☒	☐ Animals and other organisms
☒	☐ Clinical data
☒	☐ Dual use research of concern
☒	☐ Plants

Methods

n/a	Involved in the study
☒	☐ ChIP-seq
☒	☐ Flow cytometry
☒	☐ MRI-based neuroimaging

Plants

Seed stocks

Report on the source of all seed stocks or other plant material used. If applicable, state the seed stock centre and catalogue number. If plant specimens were collected from the field, describe the collection location, date and sampling procedures.

Novel plant genotypes

Describe the methods by which all novel plant genotypes were produced. This includes those generated by transgenic approaches, gene editing, chemical/radiation-based mutagenesis and hybridization. For transgenic lines, describe the transformation method, the number of independent lines analyzed and the generation upon which experiments were performed. For gene-edited lines, describe the editor used, the endogenous sequence targeted for editing, the targeting guide RNA sequence (if applicable) and how the editor was applied.

Authentication

Describe any authentication procedures for each seed stock used or novel genotype generated. Describe any experiments used to assess the effect of a mutation and, where applicable, how potential secondary effects (e.g. second site T-DNA insertions, mosiacism, off-target gene editing) were examined.
